# Differentiating atypical lipomatous tumors from lipomas with magnetic resonance imaging: a comparison with MDM2 gene amplification status

**DOI:** 10.1186/s12885-019-5524-5

**Published:** 2019-04-03

**Authors:** Carolin Knebel, Jan Neumann, Benedikt J. Schwaiger, Dimitris C. Karampinos, Daniela Pfeiffer, Katja Specht, Ulrich Lenze, Rüdiger von Eisenhart-Rothe, Ernst J. Rummeny, Klaus Woertler, Alexandra S. Gersing

**Affiliations:** 1 Department of Orthopedics and Sports Orthopedics, Technical University of Munich, Klinikum rechts der Isar, Ismaninger Strasse 22, 81675 Munich, Germany; 2 Department of Radiology, Technical University of Munich, Ismaninger Strasse 22, 81675 Munich, Germany; 3 Institute of Pathology, Technical University of Munich, Ismaninger Strasse 22, 81675 Munich, Germany

**Keywords:** Atypical lipomatous tumor, Lipoma, Magnetic resonance imaging, MDM2 amplification

## Abstract

**Background:**

To evaluate the diagnostic value of MR imaging for the differentiation of lipomas and atypical lipomatous tumors (ALT) in comparison with histology and MDM2 amplification status.

**Methods:**

Patients with well-differentiated lipomatous tumors (*n* = 113), of which 66 were diagnosed as lipoma (mean age 53 years (range, 13–82); 47% women) and 47 as atypical lipomatous tumor (ALT; mean age 60 years (range, 28–88); 64% women), were included into this study using histology and MDM2 amplification status by fluorescence in situ hybridization (FISH) as standard of reference. Preoperative MR images were retrospectively assessed by two radiologists for the following imaging features: maximum tumor diameter (mm) as well as the affected compartment (intramuscular, intermuscular or subcutaneous), septa (absent, thin (< 2 mm) or thick septa (> 2 mm) with nodular components); contrast enhancing areas within the lipomatous tumor (< 1/3 of the tumor volume, > 1/3 of the tumor volume);

**Results:**

Of the 47 patients with ALT, 40 (85.1%) presented thick septa (> 2 mm) and this finding significantly increased the likelihood of ALT (OR 6.24, 95% CI 3.36–11.59; *P* < 0.001). The likelihood of ALT was increased if the tumor exceeded a maximum diameter of 130.0 mm (OR 2.74, 95% CI 1.82–4.11, *P* < 0.001). The presence of contrast enhancement in lipomatous tumors significantly increased the likelihood of ALT (Odds ratio (OR) 2.95, 95% confidence interval (CI) 2.01–4.31; *P* < 0.001). Of the lipomas, 21.1% were located subcutaneously, 63.6% intramuscularly and 15.2% intermuscularly. On the other hand, none of the ALTs were located subcutaneously, the majority was located intermuscularly (87.3%) and a small number of ALTs was located intramuscularly (12.7%).

**Conclusions:**

Our results suggest that using specific morphological MR imaging characteristics (maximum tumor diameter, thick septa and contrast enhancement) and the information on the localization of the lipomatous tumor, a high sensitivity and substantial specificity can be achieved for the diagnosis of lipomas and ALTs.

## Background

Lipomatous tumors are the most common type of soft tissue tumors of the extremities. The majority of these tumors are atypical lipomatous tumors (ALT) or lipomas [1–3], representing 40 to 45% of the lipomatous tumors [4, 5]. ALTs may show locally aggressive growth [6–8] and even though the risk is very low, they may have the potential to metastasize or dedifferentiate [9].

In contrast a tumor is termed “well-differentiated liposarcoma” (WDL) when located in the retroperitoneum or regions (for example spermatic cord) in which the tumor cannot be resected with a sufficient surgical margin [4]. Recurrence occur more frequently due to the lack of differentiation to local adipose tissue. Histologically, ALTs consist of mature adipocytes with atypical hyperchromatic nuclei [10, 11]. These tumors often contain fibrous septa in which these atypical cells are often difficult to identify [12, 13]. Moreover, the diagnosis may be complicated by these atypical cells being scattered throughout the lesion [11], which consequently requires extensive analysis of the tumor [5]. With additional cytological characterization such as the fluorescence in situ hybridization (FISH) analysis, the presence of amplifications within marker chromosomes, e.g. in the region 12q13–15 [14–18], has been detected previously, resulting in an amplification of several genes, such as murine double minutes (MDM2), which is frequently found in ALT [5, 11, 12, 19]. A previous study has shown that MDM2 is highly sensitive for ALT and that without taking this marker into account there has been a tendency to falsely classify ALTs as lipomas in the past [12]. Previous magnetic resonance imaging studies have described that the presence of certain characteristics, such as the size of lipomatous tumors, thick septa and reduced fat content increased the likelihood of the diagnosis of ALT [13, 20, 21]. Yet, the majority of these previous studies did not include molecular genetic analysis, which have shown to be more sensitive and accurate regarding the differentiation between lipomas and ALTs [13, 20, 21]. It was previously demonstrated that lipomas were often over-diagnosed if the pathological diagnosis was based on histology only, since many lipomatous tumors that were histologically considered to be lipomas showed a positive MDM2 amplification status in the cytogenetic analysis, which is a marker highly sensitive for ALT [22].

Therefore, the purpose of this study was to assess the reliability of MR imaging criteria of ALTs and lipomas using the histopathology and the MDM2 amplification status by FISH as a standard of reference.

## Methods

### Patient selection

**Fig. 1:**
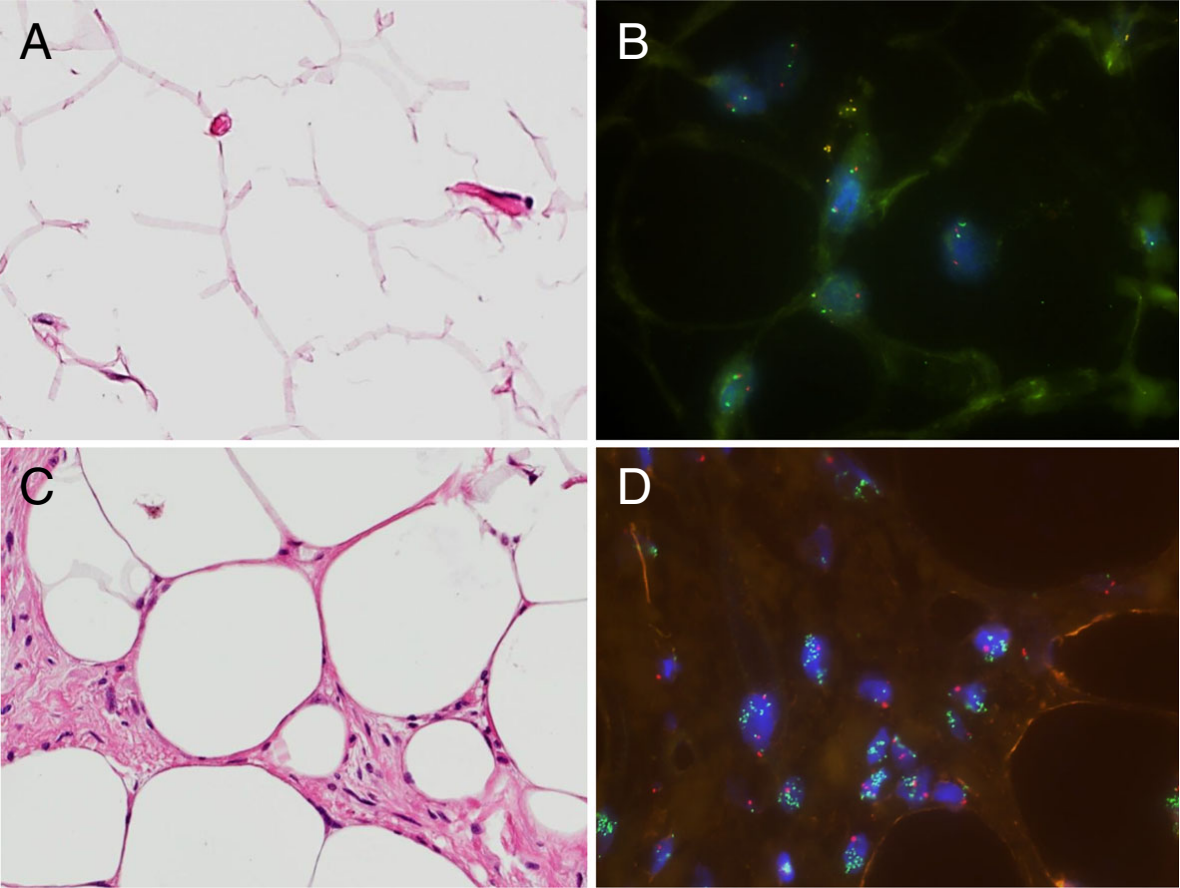
(**a**) Lipoma with equally large fat vacuoles, no atypia recognizable. (**b**) Corresponding fluorescence in situ hybridization (FISH) analysis MDM2 gene (disomy concerning MDM2, green: gen probe MDM2 region; red: centromere probe chromosome 12; two green and two red signals per cell means disomy, no amplification of the MDM2 locus = > lipoma). (**c**) atypical lipomatous tumors (ALT) with atypical stromal cells with nuclear hyperchromasia and size variations of fat vacuoles. (**d**) Corresponding fluorescence in situ hybridization (FISH) analysis MDM2 gene (Cluster-like signals in green means amplification of MDM2 locus, red signal marks the centromere probe chromosome 12 as a control = > ALT)

Institutional Review Board approval was obtained prior to this study (IRB blinded for review). Written informed consent was waived for this retrospective analysis of routinely acquired imaging and clinical data. We retrospectively reviewed the records of 272 patients with lipomatous tumors at the upper or lower extremity or trunk with surgery performed at our institution between 2010 and 2018 and histologically confirmed diagnosis of a lipoma (*n* = 206) or an ALT (*n* = 66). In all patients pre-operative MR imaging was performed. MDM2 cytogenetic status was obtained in 113 patients (Fig. 1**)** together with the histological analysis based on the World Health Organization criteria [4] after the tumor was resected. Fluorescence in situ hybridization of MDM2 gene locus (FISH). FISH analysis was performed on 4 μm-thick paraffin-embedded tissue sections following standard protocols in our laboratory of the institute of pathology using probe for centromere chromosome 12 (CEN 12) and probe for MDM2 gene locus (ZytoLight SPEC MDM2/CEN 12 Dual; Zytovision, Bremerhaven, Germany) according to the protocol provided by the manufacturer. This examination shows the chromosomal region of the human MDM2 gene as a green signal. The centromere of chromosome 12 (CEN12) is detected as a strong and intense red signal (Fig. 1). Two senior pathologists, experienced in the examination of soft tissue tumors, provided a consensus diagnosis based on the World Health Organization criteria. According to this, a final diagnosis of a lipoma was made in 66 patients and of an ALT in 47 patients. Only patients with ALT were included in this study, none of the patients showed a WDL (according to the WHO classification).

There was no significant difference regarding age and sex distribution as well as the location of the lipomatous tumor (lower limb, trunk, upper limb) between the patients that were excluded from the lipomatous tumor subgroups due to missing cytogenetic analysis and those patients that were included in the study (*p* > 0.05).

### MR protocol and image analysis

MR imaging was performed at either 3 Tesla or 1.5 Tesla scanners with various protocols. MR protocols included a T2 fast spin echo (FSE) sequence in at least two planes (e.g. axial and coronal), a short tau inversion recovery (STIR; either coronal or sagittal) sequence and an axial or coronal T1-weighted spin echo sequence with fat suppression after the administration of contrast agent.

**Fig. 2:**
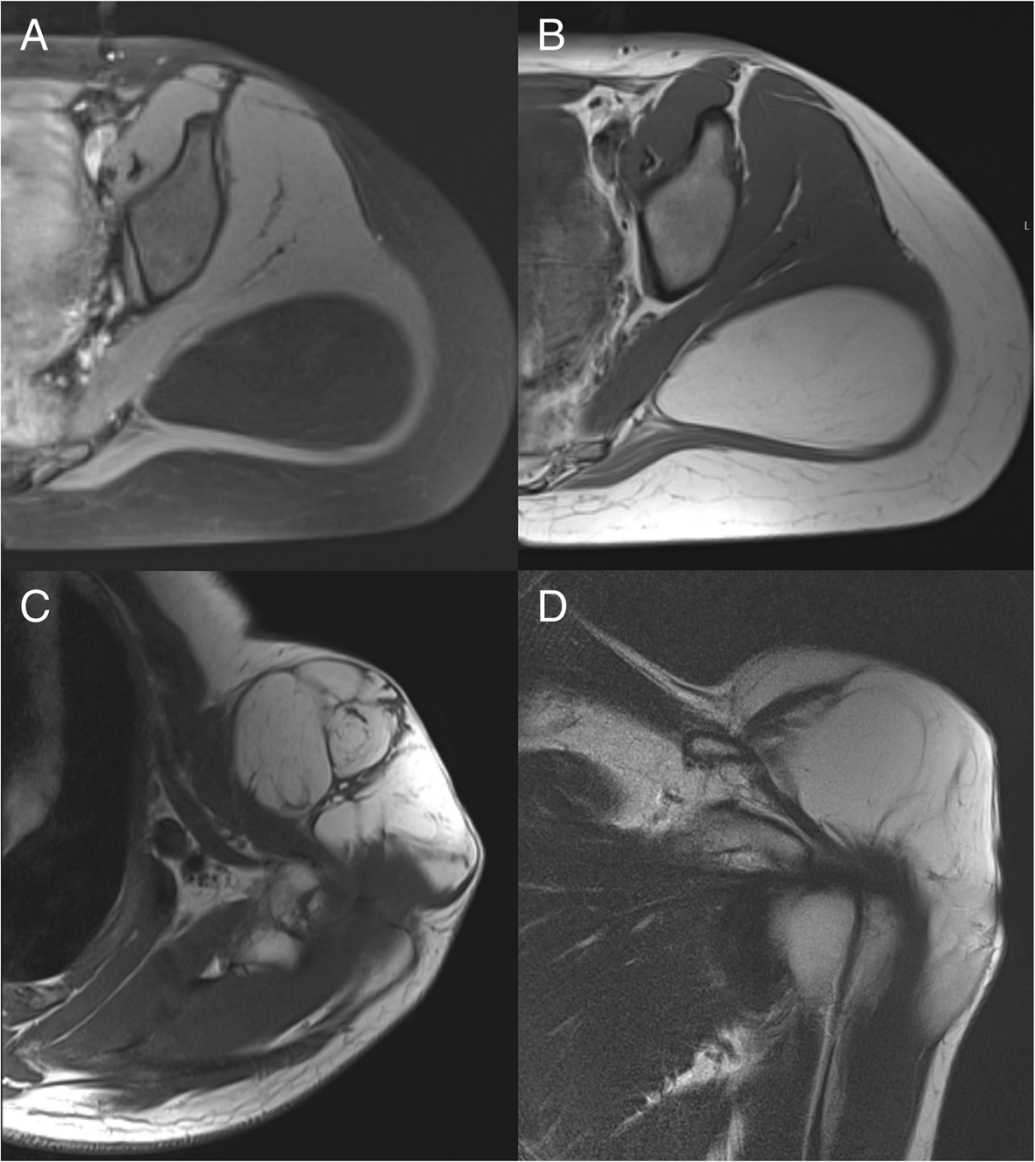
Intramuscular lipomatous tumor in a 45-year-old female patient in the left gluteus muscles. (**a**) The axial T1 weighted image with fat saturation (FS) and (**b**) the axial T2 weighted image show a well-defined lipomatous tumor which was classified as a lipoma after resection. Another 65-year-old male patient showed an intramuscular lipomatous tumor reaching into the subcutaneous region of the left shoulder. (**c**) An axial T2 weighted and a (**d**) coronal T2 weighted image demonstrating pseudo-infiltrative margins of the tumor, which was classified as an ALT after resection

**Fig. 3:**
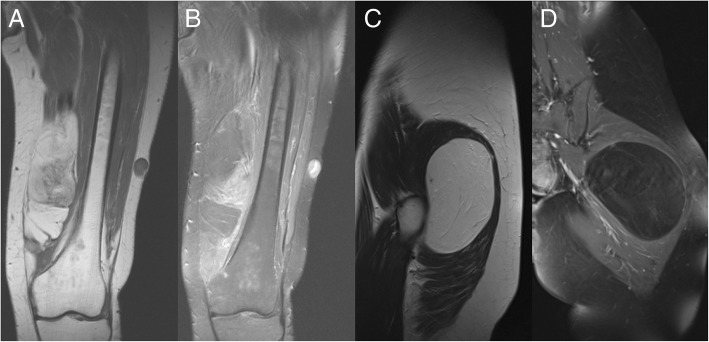
A 41-year-old male patient with a lipomatous tumor (ALT) at the medial left sided thigh showing solid, non-lipomatous components within the tumor on a (**a**) coronal T1 weighted image with (**b**) contrast enhancement (> 1/3 of the tumor volume) on a coronal T1 weighted FS image. On the other hand, there is a 45-year-old female patient showing a lipomatous tumor (lipoma) on a (**c**) sagittal T2 weighted image without (**d**) contrast enhancement on the coronal T1 weighted FS image in the gluteus region on the left side

**Fig. 4:**
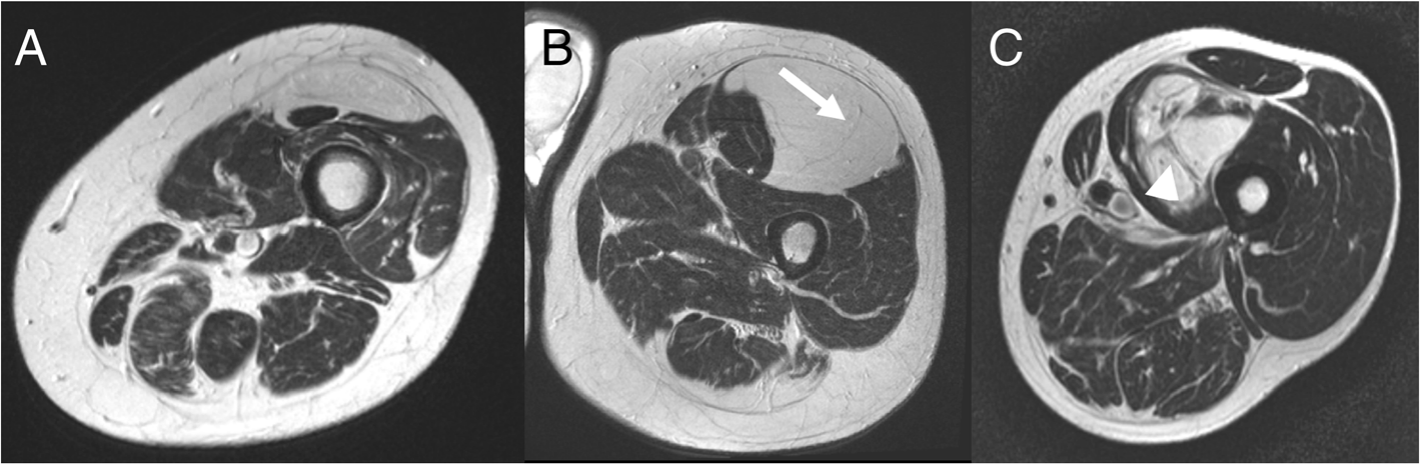
Axial T2 weighted images of lipomatous tumors (**a**) without septa; (**b**) with thin septa (< 2 mm) and (**c**) with thick septa (> 2 mm)

MR images were independently rated by two radiologists (A.S.G. and J.N.; each with 7 years of experience) blinded for clinical information including surgery and histopathological outcome parameters, using a standardized scoring sheet. The following parameters were assessed: the location of the lipomatous tumor within the body (upper limb, trunk, lower limb), as well as within the affected compartments (intramuscular, intermuscular or subcutaneous), tumor margins (well-defined or pseudo-infiltrative margins; Fig. 2), signal characteristics (predominantly fatty, mixed, predominantly non-lipomatous), contrast enhancing areas within the lipomatous tumor (< 1/3 of the tumor volume, > 1/3 of the tumor volume; Fig. 3); septa (absent, thin (< 2 mm) or thick septa (> 2 mm) with nodular components; Fig. 4); maximum tumor diameter (mm). Diagnostic image quality was rated using a four-point Likert scale (excellent, good, moderate or poor image quality) [23].

### Statistical analysis

The data were analyzed using SPSS 25.0 (IBM, Armonk, N.Y., USA) (B.J.S.). All statistical tests were performed two-sided with a level of significance (α) of 0.05.

The frequencies of MR imaging findings and demographic parameters were compared between groups with crosstabs and Pearson’s chi-squared test and Fisher’s exact test, respectively, for binary parameters. Independent samples t-tests were used for continuous and normally distributed data. Logistic regression models were used to estimate the likelihood of the presence of certain morphological features for the diagnosis of an ALT. Based on the five parameters chosen for the analysis of MR images (region, tumor size, septation, contrast enhancement, nodules) we used a univariate logistic regression model.

A receiver operating characteristic (ROC) analysis was used to assess the performance of the parameter “maximum tumor size” for the differentiation between ALT and lipoma. Youden’s J statistic was used to identify the optimal cut-off value [24].

The intra- and interreader agreement of MR imaging findings was assessed with Fleiss’ κ. For the intrareader agreement, one radiologist (initials blinded for review) repeated the readings of all patients once again after four weeks, blinded for previous results.

## Results

### Patient characteristics and tumor localization

Of 113 mature lipomatous tumors, 66 were diagnosed as lipomas and 47 as ALT using the MDM2 amplification status by FISH as standard of reference. Patients with ALTs were significantly older than patients with lipomas (median age, 60 (range, 28–88) versus 53 (13–82) years; *P* = 0.002). There was no significant difference regarding the sex distribution between the patient groups with ALT and lipoma (ALT, 63.8% women; lipoma, 47.0% women; *P* = 0.08). The majority of ALTs were located at the lower limb or at the trunk (*n* = 45 (95.7%)), whereas significantly less lipomas were located at either the lower limb or the trunk (*n* = 48 (72.7%)). Therefore, the likelihood of ALT was increased by a factor of 1.32 if tumors were located at the lower limb or the trunk (95% confidence interval 1.12–1.54, *P* = 0.002; Table 1). Of the lipomas, 21.1% were located subcutaneously, 63.6% intramuscularly and 15.2% intermuscularly. On the other hand, none of the ALTs were located subcutaneously, the majority was located intermuscularly (87.3%) and a small number of ALTs was located intramuscularly (12.7%). Margins of the of the ALTs were significantly more often pseudo-infiltrative compared to the lipoma margins (42.6% vs. 7.6%; *P* < 0.001).

**Table 1:** Frequencies, odds ratios and performance parameters for each imaging variable for patients with ALT and lipoma

**Variable**		ALT	Lipoma	Odds Ratio95% CI)^a^	*P*-value	Sensitivity^b^	Specificity^b^	PPV^b^	NPV^b^
Region	Lower limb/trunk	45	48	1.32 (1.12–1.54)	0.002	0.957	0.273	0.682	0.900
	Upper limb	2	18						
Tumor size	> 130.0 mm	37	19	2.74 (1.82–4.11)	< 0.001	0.787	0.712	0.661	0.824
	≤ 130.0 mm	10	47						
Septation	Thick (> 2 mm)	40	9	6.24 (3.36–11.59)	< 0.001	0.851	0.864	0.816	0.891
	Absent/thin (< 2 mm)	7	57						
Contrast enhancement	Presence	42	20	2.95 (2.01–4.31)	< 0.001	0.894	0.697	0.677	0.902
	Absence	5	46						
Nodules	Presence	9	0	0.81 (0.70–0.92)	0.001	0.191	1.000	1.000	0.635
	Absence	38	66						

^a^Data given as odds ratio (95% confidence interval). ^b^Sensitivity, specificity, PPV and NPV given for the identification of ALT versus lipoma, respectively. PPV, positive predictive value; NPV, negative predictive value

Of the 47 patients with ALT in this study, 42 patients were regularly followed up by MR imaging (5 patients no follow-up datas are available). The follow-up period covers an average of 29.3 months (range 4–94 months). In 7 of the 47 patients recurrence of ALT occurred after an average of 32 months (range 10–86 months): 4 patients underwent a second resection and 3 patients chose a wait-and-see approach at their own request with MRI controls. None of these 7 patients showed dedifferentiation of the liposarcoma. As lipomas are benign lesions, patients have not been routinely followed up by MR imaging. 8 patients presented in our outpatient clinic with local problem after resection (average after 13.5 months, range 5–30 months). All of them underwent MR imaging, showing a small remnant of lipoma (6 months postoperatively) in one patient. A further surgical resection was not carried out at the request of the patient. In the remaining 7 patients no local recurrence or residual lipoma has been observed.

### MR imaging criteria and image quality

**Fig. 5:**
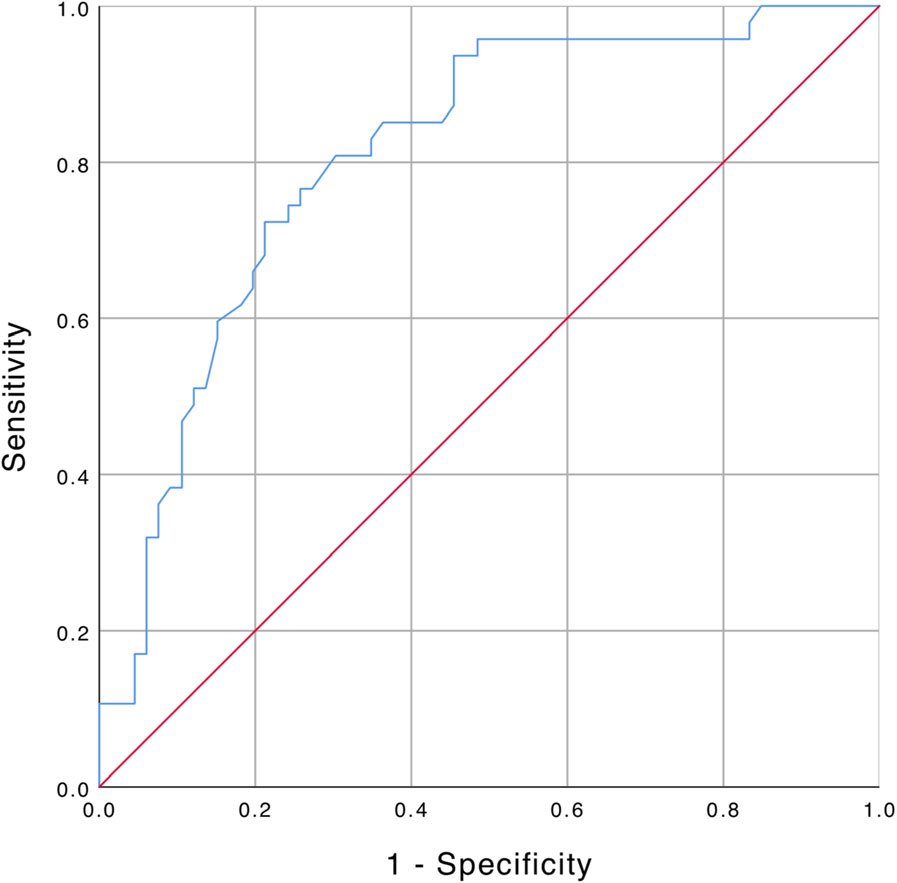
Receiver-operating-characteristic (ROC) curve demonstrating the association between maximum tumour size and entity (AUC 0.809, 95%-confidence interval 0.729–0.890)

An excellent or good image quality was achieved in 95.6% of the MR images, in 4.4% of the MR images a moderate image quality was achieved and none of the MR images received a poor image quality rating. Lipomatous tumors with a maximum tumor diameter of 130.0 mm or smaller were more likely lipomas than ALTs (82.5% vs. 17.5%; Fig. 5), whereas lipomatous tumors with a maximum diameter larger than 130.0 mm are more likely to be diagnosed as ALTs than as lipomas (66.1% vs. 33.9%; *P* < 0.001). Therefore, the likelihood of a tumor to be an ALT was increased by a factor of 2.74 if it had a maximum diameter of more than 130.0 mm (95% confidence interval 1.82–4.11, *P* < 0.001; Table 1). Tumors without contrast enhancement were more likely lipomas than ALTs (90.2% vs. 9.8%; *P* < 0.001) whereas lipomatous tumors with contrast enhancement were more likely to be diagnosed as ALTs (67.7% vs. 32.3%; *P* < 0.001). Therefore, the presence of contrast enhancement in lipomatous tumors increased the likelihood of an ALT by a factor of 2.95 (95% confidence interval 2.01–4.31; *P* < 0.001). Moreover, there was a statistical trend found showing contrast enhancement of more than 1/3 of the tumor volume in ALTs compared to lipomas (34.1% vs. 10.0%; *P* = 0.063).

Presence of nodules was seen in 9 of the 47 patients with ALT (19.1%), whereas none of the patients with lipoma presented with nodules (odds ratio 0.81 (95% confidence interval 0.70–0.92); *P* = 0.001). Of the ALTs, 17.0% presented with mixed or predominantly non-lipomatous tissue, whereas none of the lipomas showed either mixed or predominantly non-lipomatous tissue (0%; *P* = 0.001). Of the 47 patients with ALT, 40 (85.1%) presented thick septa (> 2 mm) and presence of these thick septa increased the likelihood of an ALT by a factor of 6.24 (95% confidence interval 3.36–11.59; *P* < 0.001). These thick septa were detected in only 13.6% of the lipomas.

Using the MR imaging criteria described in Table 1, the most reliable parameter was presence of thick septa (> 2 mm) with a sensitivity of 85.1% and a specificity of 86.4%. The positive predictive value was 81.6% and the negative predictive value was 89.1%. The inter-observer reliability was substantial for all criteria (κ = 0.73–0.85) and the intra-observer reliability was excellent (κ = 0.84–0.96), respectively.

In a ROC analysis of the maximum tumor size, the area under the curve (AUC) for the differentiation of the two entities was 0.809 (asymptotic 95% confidence interval 0.729–0.890), with an optimal cut-off value of 130.0 mm (*J*, 0.505; sensitivity, 0.809; specificity, 0.697). Using this cut-off value, the likelihood of a tumor to be an ALT was increased by a factor of 2.74 if it had a maximum diameter of more than 130.0 mm (95% confidence interval 1.82–4.11, *P* < 0.001; Fig. 5).

## Discussion

Our study focused on assessing MR imaging features of lipomas and ALTs in order to differentiate between the two entities, using a combination of histology and genetic testing as a standard of reference. We found that a maximum lipomatous tumor diameter of 130.0 mm or more as well as thick septa, nodules and contrast enhancement were associated with significantly higher odds of a tumor to be an ALT. Moreover, none of the ALTs were located subcutaneously. MDM2 amplification status was used in combination with the histology as a standard of reference which, to our knowledge, is the most robust pathological analysis currently available [11, 12, 22].

Previous studies have shown several imaging features to be associated with ALT: thick and nodular septa, solid non-lipomatous areas within the tumor, large tumor size [13, 20, 21]. Kransdorf et al. have described in a study with 40 MR images of patients with lipomatous tumors that imaging features suggesting malignancy are the presence of septa, the presence of nodular components and non-lipomatous mass-like components [20]. Yet, since contrast agent was administered in only eight patients in this previous study, the power of the analysis of the contrast enhancement patterns was limited. Another study showed that thick septa (defined as septa thicker than 2 mm) were more prevalent in lipomatous tumors located in deep somatic regions whereas the absence of septa or thin septa (< 2 mm) were more often found in subcutaneous lesions. In this previous study, the septa of ALTs (previously known as well-differentiated liposarcomas) showed a more prominent contrast enhancement after contrast agent administration compared to lipomas [13]. This is in line with the results of our study, in which contrast enhancement was a very strong predictor for ALTs. Yet, in this previous study, only 17 patients with ALTs were included, of whom only 10 were imaged with contrast agent administration. In another study with 12 ALTs and 48 lipomas, a score consisting of previously reported morphological features without a predictive analysis was evaluated which consisted of the following lipomatous tumor features: tumor diameter (cut-off 10 cm), the location, the presence of septa and contrast enhancement. With this score a sensitivity of 100% and a specificity of 77% was achieved. Yet, the major limitation of this study was, beside the small cohort size, that the standard of reference was inadequate due to the lack of MDM2 amplification status assessment or the assessment of other cytogenetic markers [21], and thus, several actual ALTs may have been falsely classified as lipomas.

A previous study that had assessed the reliability of MR imaging characteristics of lipomas and ALTs, diagnosed with histopathological features as well as the MDM2 amplification status showed an overall excellent sensitivity, yet a very poor specificity using the criteria assessed [12]. This may have been due to certain selected criteria such as “altered fat” signal within the lipomatous tumor, since this may have caused an over-diagnosis of ALT. Moreover, this previous study did not find the presence of septa or nodules to increase the likelihood for ALT. Since we assessed the sensitivity and specificity in our study with only the criteria showing highly significant findings in a significantly larger cohort, we were able to show a substantially higher specificity and a slightly higher sensitivity in our study compared to the previous study.

In addition, ALTs were mostly located at the lower limb and trunk, which is a finding that supports the previously reported results [12, 20]. As shown in the previous study, there were no ALTs found subcutaneously, which underlines the hypothesis that well-differentiated lipomatous tumors that are located subcutaneously are most certainly lipomas [12]. Moreover, as shown previously in other studies, there was no tendency towards a certain sex distribution, neither in the patient group with lipomas, nor in the patient group with ALTs [21, 25]. The finding that patients with ALTs were significantly older than patients with lipoma is consistent with the previous reports [4, 10, 11, 13].

This study has limitations. Even though the number of tumors assessed in this study was larger than in the previous study on lipomatous tumors and MR imaging, and the specificity of the MR imaging features assessed was substantially higher in comparison to the previous study with MDM2 as a standard of reference, yet the specificity was still not as high as the sensitivity. This may be due to the imaging appearance of certain lipomas, e.g. with regressive changes, and the consecutive over-diagnosis of ALT on MR imaging. Nevertheless, in this fairly large study group and with our statistical analysis performed, specificity was substantially higher than in the previous study that also compared MR imaging with histology and MDM2 amplification status [12]. However, we did not assess the significance of the variables (region, tumor size, septation, nodules, contrast enhancement) in a multivariate logistic regression model.

## Conclusions

In summary, our results suggest that using standard MR imaging characteristics (thick septa, maximum tumor diameter, presence of nodules and contrast enhancement), a high sensitivity and substantial specificity was achieved with a diagnosis of lipomas and ALTs in comparison to histology and MDM2 amplification status and therefore may support individual therapy selection. Moreover, none of the ALTs were located subcutaneously, they were mostly located intermuscularly and the strongest predictors of ALT were the presence of thick septa, a maximum tumor diameter of 130 mm or more and contrast enhancement.

## Abbreviations

ALT: Atypical lipomatous Tumours
FISH: fluorescence in situ hybridization
MR: magnetic resonance
WDL: well-differentiated liposarcoma
